# National inventory of emergency departments in Singapore

**DOI:** 10.1186/1865-1380-5-38

**Published:** 2012-10-31

**Authors:** Leana S Wen, Anantharaman Venkataraman, Ashley F Sullivan, Carlos A Camargo

**Affiliations:** 1 Harvard Affiliated Emergency Medicine Residency, Department of Emergency Medicine, Brigham & Women’s Hospital/Massachusetts General Hospital, Neville House, 2nd floor. 75 Francis St, Boston, MA, 02115, USA; 2 Department of Emergency Medicine, Singapore General Hospital, Outram Road, Singapore, 169608, Singapore; 3 Department of Emergency Medicine, Massachusetts General Hospital, EMNet Coordinating Center, 326 Cambridge St, Suite 410, Boston, MA, 02114, USA

**Keywords:** International emergency medicine, Emergency department classification, Emergency department utilisation, Singapore, Health policy

## Abstract

**Background:**

Emergency departments (EDs) are the basic units of emergency care. We performed a national inventory of all Singapore EDs and describe their characteristics and capabilities.

**Methods:**

Singapore EDs accessible to the general public 24/7 were surveyed using the National ED Inventories instrument ( http://www.emnet-nedi.org). ED staff members were asked about ED characteristics with reference to calendar year 2007.

**Results:**

Fourteen EDs participated (100% response). All EDs were located in hospitals, and most (92%) were independent departments. One was a psychiatric ED; the rest were general EDs. Among general EDs, all had a contiguous layout, with medical and surgical care provided in one area. All but two EDs saw both adults and children; one ED was adult-only, and the other saw only children. Six were in the public sector and seven in private health-care institutions, with public EDs seeing the majority (78%) of ED patients. Each private ED had an annual patient census of <30,000. These EDs received 2% of ambulances and had an inpatient admission rate of 7%. Each public ED had an annual census of >60,000. They received 98% of ambulances and had an inpatient admission rate of 30%. Two public EDs reported being overcapacity; no private EDs did. For both public and private EDs, availability of consultant resources in EDs was high, while technological resources varied.

**Conclusion:**

Characteristics and capabilities of Singapore EDs varied and were largely dependent on whether they are in public or private hospitals. This initial inventory establishes a benchmark to further monitor the development of emergency care in Singapore.

## Background

Emergency medicine (EM) is a relatively new specialty worldwide [1]. In Singapore, it has been a recognised specialty since 1984, and post-graduate training programmes in EM have existed since the late 1980s [2].

Prior studies have described the evolution of EM and Emergency Medical Services in Singapore [2,3]. Yet, aside from one study that attempted to classify patient complaints in the emergency department (ED), there has been no systematic study to describe or classify EDs in Singapore [4]. Even though the ED is often regarded as the basic unit of EM, little has been written to classify EDs worldwide [5]. Existing data from the National Emergency Department Inventory (NEDI)-USA, a national study of all United States EDs, suggest a large degree of heterogeneity among EDs, particularly between those in rural versus urban settings [6]. Such heterogeneity would be expected in most countries with urban and rural populations. This would also extend to the range of services available in each ED with significant variations in their annual census.

Though Singapore is one of the smallest countries in the world (only 25 miles from east to west and 15 miles from north to south and with a total population of about 5 million), there could be benefit for many to learn about the system of emergency care in what is often regarded as one of the best organised and managed countries in the world [2]. There are lessons to be learnt from each country’s EM infrastructure, from its development to its structure, its characteristics and its use of resources. Singapore is an example of a developed country with well-established medical specialties, and describing the state of EDs there can shed light on and assist other developed or developing countries of similar or different demographics and medical profiles, including those in Asia and the West. Within Singapore, there is a need for clear documentation of the capabilities and standards of service to be expected from its EDs. Specifically, there have been issues raised regarding the quality of emergency care provided in private hospitals [7]. While there is no national categorisation of levels of capabilities of EDs [8], all public health-care institutions have a service level agreement with the Ministry of Health that includes the 24-h availability of dedicated ED services. It is not clear if a similar agreement exists with all hospitals in the country. There is a need for national knowledge of the capabilities of the individual hospitals, which can form a basis for characterisation, if not categorisation, of hospital EDs. The US has led the field in formulating a system to document and inventory EDs [6]. The NEDI project has also been introduced in other countries [5]. One way forward for Singapore to improve emergency care is to collect basic and internationally comparable characteristics of all EDs (in both private and public hospitals) to benchmark locally with each other and to be able to work toward common standards.

In July 2007, the Chiefs of Singapore’s public EDs came together and agreed to participate in a national inventory project. They also urged that private hospital EDs be asked to join, which they did after a subsequent meeting in November 2007 in which consensus was reached on the need to create a Singapore ED inventory. This national inventory could potentially help each ED determine the standard of care they wish to achieve, while having an overall understanding of standards within the community. Furthermore, such an inventory can assist in further monitoring the development of emergency care in Singapore and better understand its status compared to other similar settings worldwide. Thus, the goal of our study was to perform a national inventory of all EDs in Singapore in order to describe the characteristics, resources, capabilities, and capacity of such departments in Singapore. Our hope is that such information will be useful to other countries looking to design and/or restructure their EDs, regardless of their current demographics or state of public and private sector medical care.

## Methods

This was a cross-sectional descriptive study with web-based surveys administered to either the physician-administrator or non-physician manager at each Singapore ED. Sites without Internet access were invited to participate in a paper survey. Consistent with terminology used in NEDI-USA, an ED was defined as an emergency care facility accessible to the general public that is open 24 h per day, 7 days per week [6]. A list of EDs was drawn up from all existing public and private sector hospitals in the country. This list was verified for completeness by the country coordinator, a senior emergency physician in Singapore (VA). All eligible EDs were contacted and surveyed. The study was jointly coordinated by the country coordinator and the Emergency Medicine Network (EMNet) ( http://www.emnet-nedi.org).

This study was determined to be exempt by the Institutional Review Board of Massachusetts General Hospital.

A 23-item questionnaire was employed. Participants were specifically asked about ED characteristics with reference to calendar year 2007. Survey questions were drawn, in part, from a survey that had been administered in hundreds of US EDs [6]. An initial six-question country coordinator survey also identified basic overall information on EDs and the development of the discipline in the country. Questions were subdivided into four categories: ED characteristics, ED patient characteristics, capacity, and resources and capabilities. ED characteristics included type (whether serving adults or children only, or both, or other special groups only), opening hours (whether open on all days of the week and all hours of the day, or only during selected time periods), institutional base (whether free standing or part of a hospital), and clinical independence (whether a separate clinical department within a hospital or part of another clinical department). EDs were also asked about whether they were physically contiguous or non-contiguous. A contiguous ED was defined as one in which all medical and surgical care was provided by a general ED in one unified area, with few exceptions (e.g. pregnant women in labour sent immediately to a Labour and Delivery Unit). A non-contiguous ED existed in a hospital if emergency care in that institution was provided in separate areas throughout the hospital (e.g. multi-specialty or interdisciplinary models).

ED patient characteristics referred to percentage arrivals by ambulance, length-of-stay, and proportions requiring hospital admission. ED capacity was assessed looking at the annual census, patient mix, and a subjective assessment of capacity. Resources and capabilities referred to duration of physician and consultant availability in the ED as well as the availability of resources such as computerised tomographic (CT) scans, cardiac monitors, mechanical ventilators, respiratory isolation, and computer systems to collect clinical data.

Data that required numbers were initially estimated by the respective ED administrators. Where confirmation of numbers was available to the country coordinator, such confirmation of official numbers (9 hospitals) was obtained. Otherwise, the estimates provided in the survey returns were used. These estimates were subsequently verified (5 hospitals) by the country coordinator close to the nearest thousand or hundred patients seen for the year 2007. Prior to implementation, survey questions were reviewed by members of the EMNet Steering Committee and several country coordinators. This survey has been subsequently modified for use in four other countries to profile their EDs (Additional file 1) [9].

Responses were directly downloaded from the EMNet website and then recorded into an Excel spreadsheet (Microsoft Corp., Redmond, WA). Responses received were maintained on a secure, password-protected server. Proportions were calculated using Stata 11.0 (StataCorp, College Station, TX).

## Results

### ED coverage in Singapore

All 14 EDs in Singapore participated in the survey. One other hospital was also considered for inclusion. This was not open 24/7 and was excluded from the survey. All 14 surveyed EDs operated from an institutional base, i.e. they were located in hospitals. One was a psychiatric ED, while the other 13 were general EDs. Seven EDs (including the psychiatric ED) were located in public health-care institutions that reported directly to the Ministry of Health, and the remaining seven were in the private sector. The lone psychiatric ED will be described initially, separately, and the later sections will cover the other departments surveyed.

### The psychiatric ED

The single 24-h public psychiatric ED treated both children (0 to 17 years of age) and adults and was physically part of the main psychiatric hospital. The six-bed ED was not an independent clinical department, but part of the General Psychiatry Department. It was contiguous in physical layout without a triage-to-service patient flow system. At least part of the ED was open 24/7. With an annual census of about 15,000 patients, of whom 13% were children, management of patients seen there was primarily for psychiatric disorders. Patients seen for non-psychiatric disorders would be referred immediately to one of the public general EDs in the country. Approximately 40% of all attendances came via ambulance. The inpatient admission rate for all ED visits at this institution was about 40%. This ED accounted for a major proportion of hospital admissions; about 80% of all hospital in patients were admitted through the ED. In the year 2007, this ED was regarded as working at capacity.

### General characteristics of EDs

All 13 general EDs were open to receive patients on a 24/7 basis. These EDs had a total of 287 beds, of which 92% were in public sector institutions. All private sector EDs self-reported as having less than 10 beds; all public sector EDs had more than 20. In the year 2007, public EDs managed 78% of all ED patients.

Of the 13 EDs, 11 saw both adults and children (86%), one only adults (7%) and one only children (7%). Children accounted for 24% of all ED patients. The private sector managed 29% of all children and 23% of all adults who sought care at EDs in the country.

**Figure 1:**
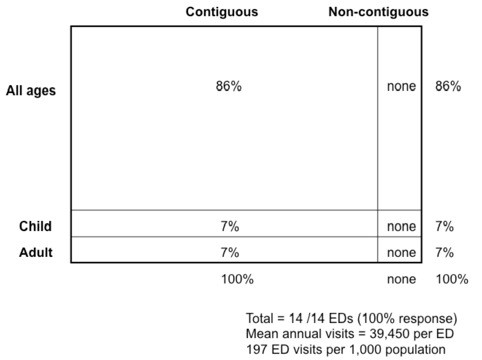
Snapshot of overall Emergency Department characteristics in Singapore.

Most (92%) were independent clinical departments (i.e. not under the jurisdiction of medicine or surgery departments). All had a contiguous layout with medical and surgical care provided in one area. All EDs except one did not have triage-to-service (e.g., triage of patients to a specific emergency service, for example, medical vs. surgical team) (Figure 1).

The median number of combined adult and child visits per year for all EDs was 39,450 (interquartile range 30,000-130,120), with a mean of 74,440. From a patient census perspective, the EDs can be divided into three groups:

a. Those managing fewer than 50,000 patients per annum. Seven EDs fell into this category, all of which were in the private sector with a mean patient load of 21,893 adults (range 12,000 to 29,738) and 8,907 children (range 1,714 to 18,000).

b. Those managing between 50,000 to 100,000 patients per annum. There was a single public sector ED that reported 67,211 adult and 1,752 children visits.

c. Those managing more than 100,000 per annum. All five of these were public sector EDs. There were four adult hospitals, three of which saw adults and children with a mean annual adult census of 120,464 (range 89,730 to 141,032) and a mean children attendance of 11,954 (range 4,664 to 18,934). The adult-only public sector ED saw 155,786 patients that year. The one public children’s ED had an annual census of 130,119.

### ED patient characteristics

Unlike the psychiatric ED, every other ED answered that 20% or less of their patient population arrived by ambulance, with 62% reporting that 2% or less were brought by such vehicles. Of these latter, all were private sector EDs except for one, which was a large public sector children’s ED. Nearly 98% of all ambulance patients were sent to public sector hospitals.

Approximately half of EDs (45%) reported that >25% of ED visits led to admission; all except one (92%) reported that >10% of ED visits led to admission (Table 1). For the public sector institutions, the mean inpatient admission rate from the ED was 30%. This was markedly different from the inpatient admission rate from private sector EDs, which averaged 7%. Overall, 6% of all patients requiring admission through EDs came from private institutions; 94% of those requiring admission came from public sector EDs.

**Table 1:** **Characteristics of general emergency departments (ED) in Singapore (*n *= 14, 100% response rate)**

	**Proportion or median**
*General characteristics*	
Hospital based	100%
Independent department	92%
Contiguous	100%
Annual ED visits (median)	39,450
*Patient experiences in ED*	
Percentage of ED patients arriving by ambulance	
≤20%	100%
Length of stay	
<1 h	23%
1-6 h	77%
>6 h	0%
Percentage of ED visits leading to admission	
>25%	45%
>10%	92%
*Resources and capabilities*	
Physician in ED 24/7	100%
Dedicated CT scanner	46%
Cardiac monitor	92%
Mechanical ventilator	61%
Respiratory isolation (negative-pressure room)	54%
Computer system to collect clinical data	69%

Note: All 14 hospitals answered 100% of the survey questions completely. There were no instances of any questions in the survey being left unanswered.

Patient length of stay in the ED was variable. The majority of EDs (77%) reported an average length of stay of between 1 and 6 h. Three EDs (23%) reported an average length of stay of less than 1 h, with all these being private EDs. No ED reported an average length of stay of over 6 h. Computation of length of stay did not include patients who were admitted to protocol-based ED observation units. The ED appears to be a major route of hospital admissions for public sector hospitals, with all six reporting that the ED contributed to over 50% of all hospital admissions (range 55% to 90%). Two of the seven private medical institutions reported that their EDs contributed 5% and 18%, respectively, of overall hospital admissions. The other five private EDs were unable to report theses statistics.

### Capacity

A majority of respondents (70%) considered their ED at capacity or at good balance. Only 15% reported their ED as being undercapacity and 15% as overcapacity. Amongst public hospitals, two were at overcapacity, three were at capacity or at good balance, and one felt they were at undercapacity. The two reporting overcapacity both had an annual census in excess of 130,000 and inpatient admission rates in excess of 30%. Amongst private institutions, six responded to be at capacity or at good balance and one was undercapacity. None of the private sector EDs considered themselves to be at overcapacity.

### Resources and capabilities

**Figure 2:**
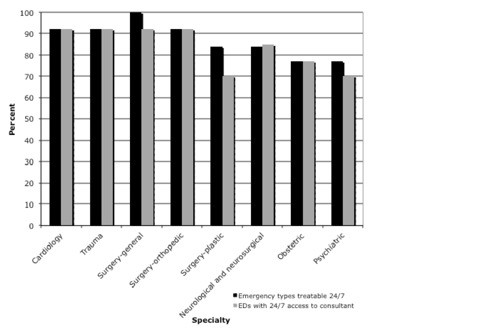
Association between emergencies identified as treatable and availability of consultants in the emergency departments, by type of emergency.

All EDs were staffed 24/7 by physicians. Most emergency types could be treated 24/7 in the EDs (Table 2). However, dental, obstetric, gynaecological and maxillofacial consultants were not available in two of the large public general hospitals and in at least two of the private institutions. Psychiatric care was also not available in at least three private institutions. All public hospital EDs and one of the seven private institutions had 24-h anaesthesia coverage within 30 min of call. For the other six private institutions, one would have an anaesthetist available between 30 and 60 min after call and the others after 60 min. Plastic surgery consultants would have been available immediately in two public and one private institution. The availability of consultants appeared to correlate with the type of emergency the EDs reported that they were capable of treating (Figure 2). Every one of the institutions surveyed would have had at least one nurse on duty in at least one of the ED areas, 24 h a day and 7 days a week.

**Table 2:** **Emergency types identified as treatable in emergency departments in Singapore (*n *= 14, 100% response rate)**

**Emergency Type**	**Example of emergency**	**Percentage of EDs able to treat 24/7 (%)**
Medical-Oncology	Fever and neutropenia	100%
Medical-Other	Urinary tract infection, acute asthma	100%
Urological	Kidney stone	100%
Surgical-General	Acute appendicitis, pneumothorax	100%
Medical-Cardiology	Arrhythmia, acute myocardial infarction	92%
Trauma	Motor vehicle crash, gun shot wound	92%
Ear, Nose, Throat	Severe epistaxis	92%
Ophthalmological	Acute glaucoma, eye injury	92%
Toxicological	Overdose, carbon monoxide poisoning	92%
Surgical-Hand	Tendon injury	92%
Surgical-Orthopaedic	Long bone fractures	92%
Neurological and Neurosurgical	Acute thromboembolic stroke, intracranial haemorrhage	84%
Gynaecological	Ruptured ovarian cyst, yeast infection	84%
Surgical-Plastic	Severe lip laceration	84%
Obstetrical	Complications of pregnancy	77%
Psychiatric	Psychosis	77%
Dental	Tooth extraction	69%
Surgical-Oral maxillofacial	Jaw fracture, oral abscess	62%

Note: All 14 hospitals answered 100% of the survey questions completely. There were no instances of any questions in the survey being left unanswered.

Technological support was variable. Virtually every ED (92%) had cardiac monitoring available immediately. Six (three in the public sector and three in the private sector) of the 13 EDs had a dedicated CT scanner. All public hospitals and two private sector institutions had mechanical ventilators available immediately in the ED. Five of the six public hospitals and three of the seven private facilities had respiratory isolation (negative pressure) rooms available in the ED. While all public hospitals had computer systems to collect clinical data in the ED, four private EDs were still dependent on manual systems for such information collection (Table 1).

## Discussion

There is variability in the characteristics and capabilities of EDs in Singapore. The differences appear to be dependent primarily on whether they are in public or private sector institutions.

Similarities amongst EDs in Singapore appear to be for patient census (> 20,000 per annum), which is considered reasonable by most countries elsewhere, a contiguous layout with medical and surgical emergencies seen in one area, being open and accessible to the public 24/7 and generally having access to a wide range of medical specialists across the various hospitals.

Differences amongst Singapore EDs appear to be primarily between those in the private versus the public sectors in terms of patient census (30,000 or less patients per annum in private sector hospitals versus at least 60,000 patients in public hospitals, which has major implications on issues such as floor space, size of facility and trained manpower required to manage the volume load in public EDs), staffing by emergency medicine board-certified physicians and emergency trained nursing staff (some of whom have also undergone specialty training in Emergency Nursing), ED bed complement and inpatient admission rates (10% or less in the private sector compared to the generally greater than 25% in the public sector). This may reflect a higher patient acuity in the public sector population. As a result of a series of public education programs conducted in public institutions over the previous 2 decades, and other social initiatives, there has been a significant shift in the case mix of public sector EDs in Singapore such that patients with low-acuity complaints generally do not visit public EDs [10]. Such a shift would be likely to result in higher acuity patients being seen, resulting in more time spent on patient care per patient seen and a longer stay in the ED prior to a decision on admission or discharge. Length of stay also appeared to be generally much shorter in private EDs than public ones. This may be owing to a lower patient acuity or lower overall census in private EDs, or it may suggest an extremely rapid process of care in these private institutions.

**Figure 3:**
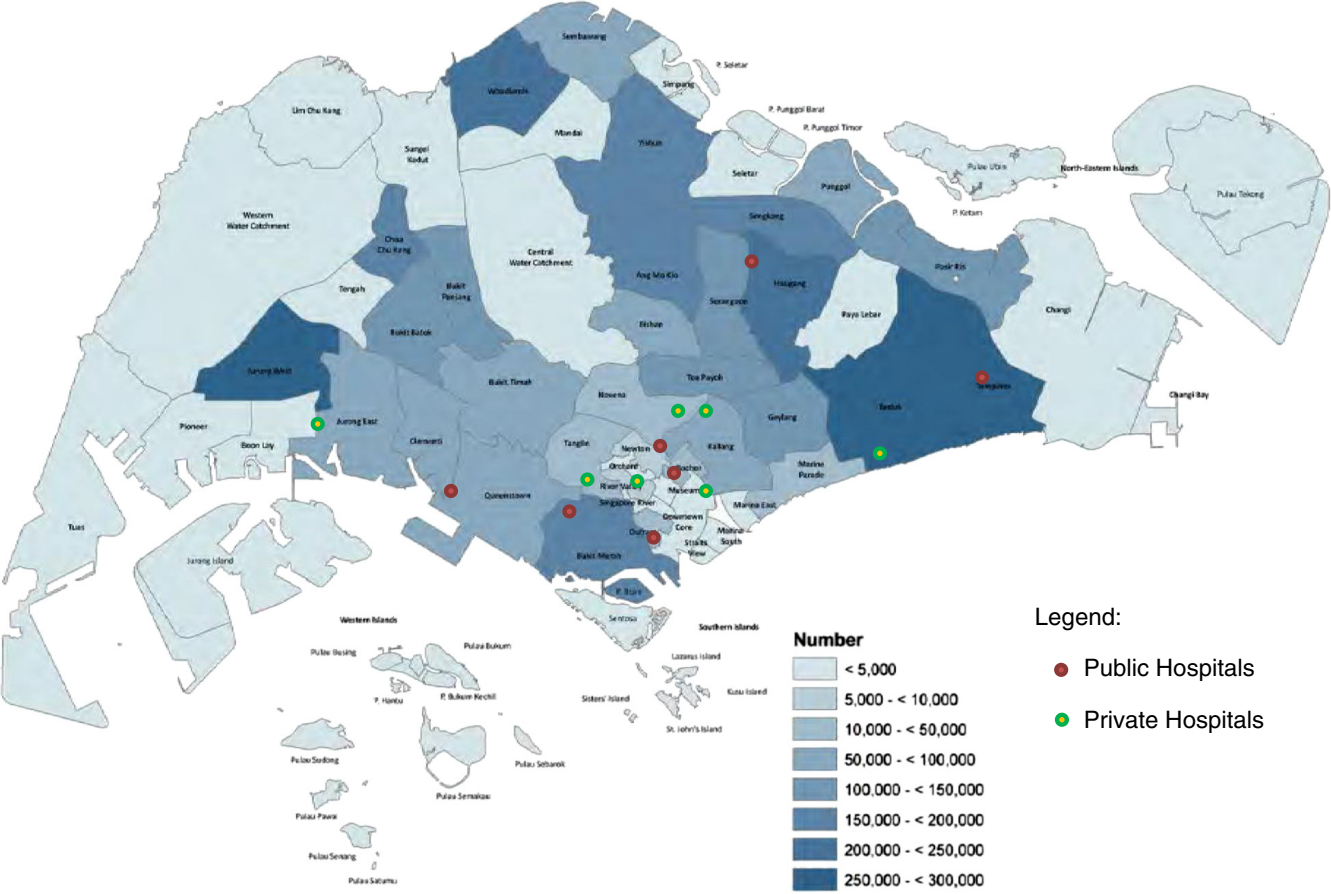
Geographical map of Singapore with emergency department locations marked.

There is currently no congregation of public or private hospitals in locations of particular economic or social status. Being a very small and compact country, the distribution of hospitals is fairly even in relation to the population spread and is generally not seen as particularly influencing demand in specific individual hospitals (Figure 3).

Taken together, our data would suggest that if one were to characterise public sector EDs in Singapore, they could be described as having a large number of beds; large numbers of high-acuity patients with a significant inpatient admission rate that contribute a major proportion of hospital inpatients; high public emergency ambulance coverage; staffing by emergency medicine board-certified physicians; being well-equipped with monitors, ventilators and respiratory isolation units; and making reasonably good use of information technology. This would place Singapore public sector EDs as being equivalent to some of the best similar facilities in countries such as the US [6].

Conversely, our data suggest that private sector EDs may be seen as having small numbers of beds with a relatively low-acuity patient population in significantly smaller numbers that contribute a small proportion of hospital inpatients and that is seldom serviced by emergency ambulances. In addition, such private EDs appear to be staffed by doctors who utilise technology more sparingly in the daily care of patients. This lower technology care provided may not truly reflect the good work that is carried out in private sector EDs. These private EDs have been able to demonstrate shorter turn-around times, which may reflect greater efficiencies in process management and more judicious use of resources (albeit with lower patient volumes and likely lower acuity). A conclusion about the quality of emergency care cannot be made because this study, after all, did not compare patient outcomes.

A future study may be useful in examining quality differences between private and public EDs. One of the consequences of being perceived as a low-performance ED may be a greater reluctance on the part of public emergency ambulance services and patients to want to use them for emergency care. In addition, national emergency and disaster planners may doubt the ability of smaller private sector EDs, and hence, private hospitals, to be able to handle mass casualties and major disease outbreaks. This would be doing a major disservice to the large number of excellent physicians who had previously been captains of the profession in the public sector and have subsequently moved to private sector institutions so as to manage a different lifestyle. At the same time, it is important to channel resources where they are most needed and to ensure that every ED is held to a similar national standard, no matter if it is publicly or privately operated.

There is a need to examine why the wide variability exists between public and private sector EDs in Singapore and how this variability affects patient care and patient outcomes. This survey was not structured to examine such reasons. Since the data were collected with reference to the year 2007, it is possible that some of the differences have been reduced over the last few years. For public hospitals, we know that one new major hospital has begun operations in 2010 and is managing a reasonably large patient load in the moderate range. The distribution of information and other technology could have evened out over the last few years. Anecdotally, emergency medicine board-certified physicians used to only be at public-sector hospitals, but in recent years have begun gradually moving into private sector EDs. Our national inventory should be repeated at regular intervals and also include quality indicators for this to be benchmarked and then set targets for future improvement.

There are other notable findings. One is the relatively low length of stay, accompanied by the finding of only two EDs reporting themselves to be overcapacity. EDs in other developed countries have been struggling with issues related to overcrowding [11]. Singapore faced a similar problem, and, in recent years, has implemented multiple health-care system interventions and public education programmes to reduce ED crowding and optimise ED utilisation for emergency complaints. A study in 2008 found that over a 12-year period following the implementation of these interventions, non-emergent use of the ED had fallen from 57% to 12% [10]. That the interventions have had their intended impact on reducing non-emergency attendances is suggested by our finding that only two EDs self-reported to be overcapacity. The concern about overcapacity may, however, reflect access block issues in these institutions. These system interventions can still be instructive to other countries, such as the US, that experience severe overcrowding in their urban EDs [11].

Another finding is that technological resources are generally available. It is interesting to note the evolution of technology in Singapore EDs. Cardiac monitoring facilities had been available in most EDs for many years. These were initially mainly in the form of stand-alone ECG monitors placed next to patients while awaiting transfer to other areas of hospitals. In the 1990s, dedicated, fixed ECG monitors were made available in all resuscitation bays of the largest hospital and gradually extended to all public EDs in the country. Telemetry was also gradually introduced to public EDs in the 1990s. A seven-bedded chest pain unit was started at one of the EDs in 1998. Such units are now available in at least three public EDs as part of emergency observation units.

A CT scanner was initially available within only one ED in the 1990s. The 2000s saw the use of hospital CT scanners being made available to all public EDs. Currently, at least six EDs have CT scanners either within or just adjacent to their premises. In some hospital EDs, residents discuss the patient with a consultant with admitting privileges to the institution and then proceed to make arrangements with the radiology service for such a resource to be made available to the patient.

Mechanical ventilators are available in the resuscitation areas of all public EDs as part of national disaster preparedness. This relates to public perception for the need for prompt management of disaster casualties, especially after the multiple terrorist incidents that have occurred on the international scene over the last 1 ½ decades and the increasing reporting of major disasters such as earthquakes, tsunamis and technological disasters. These EDs have learnt to become familiar with the use of ventilators in the management of sick patients on a daily basis.

Respiratory isolation facilities have become available in public EDs and in a few private institutions after the national experience with the outbreak of the Severe Acute Respiratory Syndrome in 2003. Such units are not readily available in most EDs in the region or even in Asia, except in communities keenly aware of the need for such facilities to contain, isolate and minimise the spread of communicable diseases brought into the department by infected patients. The absence of critical services or equipment in particular would usually mean that patients would not be denied these services, but that secondary referrals or transfers to facilities providing such services would be arranged.

EM is developing around the world, and Singapore is among the few countries that have clearly developed and established the specialty of EM for over 2 decades. Leaders in Singaporean medicine have suggested that Singapore has a role in promoting global health [12]. Indeed, Singapore is well poised from a number of different perspectives to influence international health development, especially since it has an efficient health-care system that provides high-quality universal health care while spending just 3-4% of its gross domestic product on health [13]. Our findings about the advanced and organised nature of EDs in Singapore also provide support for Singapore having a major role in the advancement of EM. For example, it has been suggested that resource-poor settings may benefit from preferential training of emergency physicians capable of treating all emergencies [14]. For countries with nascent emergency care systems, Singapore could offer a useful model for EM workforce studies, residency training curriculum, and ED benchmarking and comparisons. Countries that have a strong private sector may also learn from the Singapore experience when it comes to coordinating and comparing delivery of care by public and private EDs.

Our study has several potential limitations. We recognise that this is an initial study with descriptive statistics, but it provides new information to guide efforts to advance emergency care in Singapore. The survey also did not examine quality of care issues, specifically the quality of care between EDs in pubic and private sectors. Such a comparison is very important, but would have required a different format of investigation and would have had to be prospective over a significant time period. Such a study needs to be conducted at intervals of at least 5 years, with benchmarking of targets to be achieved in specific instances.

Another limitation is that the survey used is not validated. To our knowledge, a validated instrument to assess EDs worldwide does not exist. Questions from our survey have been used in studies of US EDs and also have been used successfully in several other countries, ensuring usability and that the wording of questions was appropriate for diverse contexts [6,9].

In addition, there is the limitation that this study relies on self-reported data. ED administrators were asked to supply data when available. When exact figures were unavailable, ED physician-administrators provided their closest approximation. As the survey was anonymous, with 100% participation, we do not suspect a systematic bias in the responses.

## Conclusions

This first study to characterise EDs in Singapore helps to establish a baseline measure of emergency care for the year 2007. We hope that our study will assist in further monitoring and improving emergency care in Singapore, as well as promoting the development of EM in other settings worldwide.

## Abbreviations

EM: Emergency medicine; ED: Emergency department; NEDI: National emergency department inventory.

## Competing interests

The authors state that we have no competing interests.

## Authors’ contributions

LSW is coordinating the NEDI-international manuscript submissions at EMNet. She conducted background research, performed data analysis and drafted the manuscript. VA was the country coordinator who oversaw data collection. He contributed substantially to data interpretation and to the manuscript writing. VA takes responsibility for the paper as a whole. AFS helped to design the survey instrument used and assisted with data analysis. She contributed substantially to the manuscript revision. CAC conceived the study, wrote the study protocol, designed the survey instrument, assisted with data analysis and contributed substantially to the manuscript revision. All authors read and approved the final manuscript.

## Supplementary Material

Additional file 1 **Survey instrument.** The survey questionnaire was divided into four major branch points leading to ten different surveys with content tailored to the specific type of emergency department (ED). The major branch points are: (1) contiguous vs. non-contiguous ED, (2) triage to service vs. no triage to service, (3) open 24/7 vs. not open 24/7 and (4) 1 leader vs. >1 leader. The compiled data from the surveys were subsequently combined into a single data set. Included is a sample survey for a contiguous ED without triage to service, 24/7, and one leader. Click here for file
