# Supplementary material for: National inventory of emergency departments in Singapore
Source: Int J Emerg Med. 2012 Oct 31;5:38. doi: 10.1186/1865-1380-5-38 (PMC3518169; doi:10.1186/1865-1380-5-38)
Supplement: Additional file 1 — Survey instrument. The survey questionnaire was divided into four major branch points leading to ten different surveys with content tailored to the specific type of emergency department (ED). The major branch points are: (1) contiguous vs. non-contiguous ED, (2) triage to service vs. no triage to service, (3) open 24/7 vs. not open 24/7 and (4) 1 leader vs. >1 leader. The compiled data from the surveys were subsequently combined into a single data set. Included is a sample survey for a contiguous ED without triage to service, 24/7, and one leader. [file 1865-1380-5-38-S1.doc]

**International Emergency Department Survey**

The NEDI project is conducted by the Emergency Medicine Network (EMNet), an emergency medicine research group, to better understand the characteristics and scope of Emergency Departments (EDs) internationally.

Since unplanned medical problems affect people worldwide, NEDI aspires to describe emergency healthcare systems around the world. The NEDI online survey identifies similarities and differences in EDs with a philosophy that different emergency healthcare systems can learn from one another. Using technology to facilitate cooperation, we hope that NEDI will serve as a platform for exchanging ideas on improving the accessibility and quality of emergency care for all.

If you work clinically in an ED, please proceed with the survey.

Are you willing to provide descriptive information about your Emergency Department?

Yes

No

🡪 If NO, please save this survey and return to it when you are ready to fill it out. Thank you for your time.

Please answer the following questions with estimates for the year 2008(unless otherwise indicated).

1. Is your ED physically part of a hospital (e.g. same building or adjacent):

Yes  No

🡪 If no, please specify the physical location or structure of the ED (e.g. urgent care center, mother and child center, community health center):

2. Please estimate the total number of hospital beds; enter 999999 if unknown:

3. Please estimate the percent of your patients that are children; enter 999999 if unknown:

4. Is the physical plant of your ED:

**Contiguous** – all medical (e.g. heart attack, acute asthma) and surgical (e.g. trauma) emergency care provided by a general ED in one unified area.

OR

**Non-contiguous** – emergency care provided in separate areas throughout the hospital; i.e. medical emergency care (e.g. heart attack, acute asthma) provided in a geographically distinct area from surgical emergency care (e.g. trauma).

5. Is there a triage to service (i.e. triage of patients to a specific emergency service, e.g. medical vs. surgical team)?

Yes  No

6. Please, indicate the total number of ED beds, enter 999999 if unknown:

7a. In 2008, was your ED open 24 hours/day, 7 days/week?

Yes  No

7b. Who runs the emergency department? (Note: If the first OR second OR last answer is checked, a respondent went down the "1 leader" path.)

ED director who oversees all emergency services (e.g. medicine and surgery)

Each service has its own director, however, they jointly oversee the emergency department (e.g. have board meetings).

Emergency services share the same hospital area but are otherwise completely independent of one another, each with its own leadership.

Other, please specify:

8. Please indicate whether the following types of emergencies can be treated in your ED and whether treatment is available 24/7:

|  | No | Yes | Not Available 24/7 | Available 24/7 |
| --- | --- | --- | --- | --- |
| a. Medical- Cardiology (e.g., arrhythmia, acute myocardial infarction) |  |  |  |  |
| b. Medical- Oncology (e.g., fever and neutropenia) |  |  |  |  |
| c. Medical- Other (e.g., urinary tract infection, acute asthma) |  |  |  |  |
| d. Trauma (e.g., motor vehicle crash, gun shot wound) |  |  |  |  |
| e. Neurological and neurosurgical (e.g., acute thromboembolic stroke, intracranial hemorrhage) |  |  |  |  |
| f. Urological (e.g., kidney stone) |  |  |  |  |
| g. Obstetrical (e.g., complications of pregnancy) |  |  |  |  |
| h. Gynecological (e.g., ruptured ovarian cyst, yeast infection) |  |  |  |  |
| i. Ear, Nose, Throat (e.g., severe epistaxis) |  |  |  |  |
| j. Ophthalmological (e.g., acute glaucoma, eye injury) |  |  |  |  |
| k. Toxicological (e.g., overdose, carbon monoxide poisoning) |  |  |  |  |
| l. Psychiatric (e.g., psychosis) |  |  |  |  |
| m. Dental (e.g., tooth extraction) |  |  |  |  |
| n. Surgical- Oral maxillofacial (e.g., jaw fractures, oral abscesses) |  |  |  |  |
| o. Surgical- Plastic (e.g., severe lip laceration) |  |  |  |  |
| p. Surgical- Hand (e.g., tendon injury) |  |  |  |  |
| q. Surgical- Orthopedic (e.g., long bone fractures) |  |  |  |  |
| r. Surgical- General (e.g., acute appendicitis, pneumothorax) |  |  |  |  |

9. Please indicate the annual number of patient visits at your ED in 2008:

Children (e.g. ages 0-17) =        Unknown

Adults (e.g. ages 18+) =        Unknown

Total (Adults + Children) =        Unknown

If your ED uses a different cut off age to distinguish between children and adults, please specify:       years

10. Approximate percent of patients that arrived at your ED by ambulance:

< 20%

20 – 39%

40 – 59%

> 60 – 79%

80% or higher

Unknown

11. Do patients require a referral from a physician, or need to arrive by ambulance, in order to receive emergency care? (i.e., with rare exceptions, patients without a referral are not seen)

Yes  No

12. What is the average length of stay at your ED?

< 1 hour

1-6 hours

> 6 hours

13a. Is a physician available to the ED, 24 hours/day, 7 days/week?

Yes—A physician is physically present in the ED, 24/7

Yes—A physician is available to the ED from within the hospital, 24/7

No—Variable staffing (e.g., a physician is available to the ED from outside the hospital)

13b. Is at least one nurse on duty in the ED, 24 hours/day, 7 days/week?

Yes  No

14. Are the following consultants available in-person to the ED? Residents or registrars qualify. If YES, please indicate, on average, how long it takes the consultant to arrive and whether the consultant is available 24/7:

|  | No | Yes 0-29 min | Yes 30-59 min | Yes ≥60 min | Not available 24/7 | Available 24/7 |
| --- | --- | --- | --- | --- | --- | --- |
| a. Anesthesiologist |  |  |  |  |  |  |
| b. Cardiologist |  |  |  |  |  |  |
| c. General Surgeon |  |  |  |  |  |  |
| d. Neurologist |  |  |  |  |  |  |
| e. Neurosurgeon |  |  |  |  |  |  |
| f. Obstetrician-Gynecologist |  |  |  |  |  |  |
| g. Orthopedic Surgeon |  |  |  |  |  |  |
| h. Plastic Surgeon |  |  |  |  |  |  |
| i. Psychiatrist |  |  |  |  |  |  |

15. In 2008, how would you describe your hospital’s ED?

Under capacity

Good balance

At capacity

Over capacity

16. Please respond “yes” or “no” in reference to the following questions:

|  | No | Yes |
| --- | --- | --- |
| a. Is there a dedicated CT scanner for the ED (i.e. preferentially for ED patients)? |  |  |
| b. Is there a cardiac monitor available immediately in the ED? |  |  |
| c. Is there a mechanical ventilator available immediately in the ED? |  |  |
| d. Is there a respiratory isolation (negative pressure) room available in the ED? |  |  |
| e. Is a computer system used to collect clinical data in the ED? |  |  |
| f. Is internet access available in the clinical area of the ED? |  |  |
| g. Is there a clinical laboratory available for your ED with the capacity to perform a potassium blood test 24/7? |  |  |

17a. Approximate percent of your hospital’s total admissions (i.e. inpatient hospitalizations) that were admitted through the ED:

< 20%

20 – 39%

40 – 59%

60 – 79%

80% or higher

Unknown

Question does not apply; ED is not part of the hospital

17b. Approximate percent of ED visits that led to admission (i.e. inpatient hospitalization), including patients admitted to observation unit/status (*in some facilities, a short-stay in the “observation unit” or an admission is designated as “observation status” if the length of stay is < 24 hours*):

< 20%

20 – 39%

40 – 59%

60 – 79%

80% or higher

Unknown

Question does not apply; ED is not part of the hospital

18. Is the ED an official department within the hospital organization (e.g. separate and independent from the Departments of Medicine or Surgery with its own leadership and staff)?

Yes  No

🡪 If NO, ED is part of which department (specify):

Please include any comments that would help us understand your survey responses. (e.g., if your ED serves a specific patient population or is a specialty ED):

Thank you for taking the time to complete this questionnaire!
